# Congenital sebaceous choristoma of the tongue: A rare case report

**DOI:** 10.4317/jced.59451

**Published:** 2022-05-01

**Authors:** Letícia-Côgo Marques, Luiza-de Moura-Carvalho Figueira, Lilian Almeida, Rafaela-Elvira Rozza-de-Menezes, Arley Silva-Junior, Karin-Soares Cunha, Danielle-Castex Conde

**Affiliations:** 1 DDS, MSc. Postgraduate Program in Pathology, School of Medicine, Universidade Federal Fluminense (UFF), Niterói, RJ, Brazil; 2 DDS, PhD. Postgraduate Program in Pathology, School of Medicine, Universidade Federal Fluminense (UFF), Niterói, RJ, Brazil

## Abstract

The most common oral choristomas are consisted of thyroid tissue and bone. The presence of sebaceous glands in the oral mucosa, especially in the buccal mucosa and labial mucosa, is often considered a normal anatomical variation since they are observed in about 80% of the population and are called ectopic sebaceous glands or Fordyce’s granules. However, the presence of these glands on the tongue is rare, with only 11 cases in the dorsum of the tongue reported in the English literature, and it is considered a choristoma. This paper aims to report the third case in the literature of a congenital sebaceous choristoma on the tongue. An 11-year-old white male patient presented a firm sessile papule, without color alteration, measuring 0.4 cm x 0.3 cm in diameter, in the middle third of the dorsum of the tongue with a slight increased size in the last months. Histopathological examination showed an invagination of the epithelium into the connective tissue, forming a ductal structure covered by stratified squamous epithelium. The deeper areas had normal well-differentiated sebaceous glands, with ducts connected to the central duct. Considering clinical and histopathological findings the diagnosis was sebaceous choristoma. Despite being rare, sebaceous choristomas should also be considered in the differential diagnosis of tongue abnormalities or lesions. Although the pathogenesis is not well understood, the present report, as a congenital choristoma case in the midline, reinforces the hypothesis of a disorder with embryological origin and a possible relationship with thyroglossal duct remnants.

** Key words:**Choristoma, Oral Mucosa, Tongue.

## Introduction

The entities hamartoma and choristoma were introduced by Albrecht in 1904 to differentiate malformations from true neoplasms (1). Hamartoma is an overgrowth of mature tissue native to the organ of origin (2). Choristoma is a growth of normal tissue in an abnormal location (3-6). The pathogenesis of choristoma is uncertain (5-7), but the complexity of the orofacial region embryogenesis, such as the fusion of the maxillary processes with the palatal processes, can result in transference and displacement of cell nests and heteroplasia (8).

A wide variety of choristomas have been described in the oral mucosa, such as salivary, cartilaginous, bone, thyroid, epidermal, glial, and gastric gland choristoma (3,4,6). In a review by Chou *et al*. (3), the most common oral choristomas are those consisting of thyroid tissue and bone.

The presence of sebaceous glands in the oral mucosa, especially in the buccal mucosa and labial mucosa, is often considered a normal anatomical variation (5) since they are observed in about 80% of the population and are called ectopic sebaceous glands or Fordyce’s granules (3,9,10). However, the presence of these glands in the tongue is rare and is considered a choristoma (4,5).

Although Fordyce’s granules have few diagnostic problems, choristomas consisting of sebaceous glands are rarely considered in the differential diagnosis of tongue alterations or lesions (7). This paper aims to report the third case in the literature of a congenital sebaceous choristoma, in an 11-year-old patient, located in the dorsum of the tongue.

## Case Repor

An 11-year-old white male patient was referred by the pediatric dentistry service to the Oral Diagnosis Outpatient Clinic for evaluation of a congenital tongue lesion, which was asymptomatic and with a slight increase in size in the last months. Past pathologic history included urinary tract infection (1-year-old), conjunctivitis (2 years old), chickenpox (3 years old), sty (9 years old), and recent food allergy to instant noodles.

**Figure 1:**
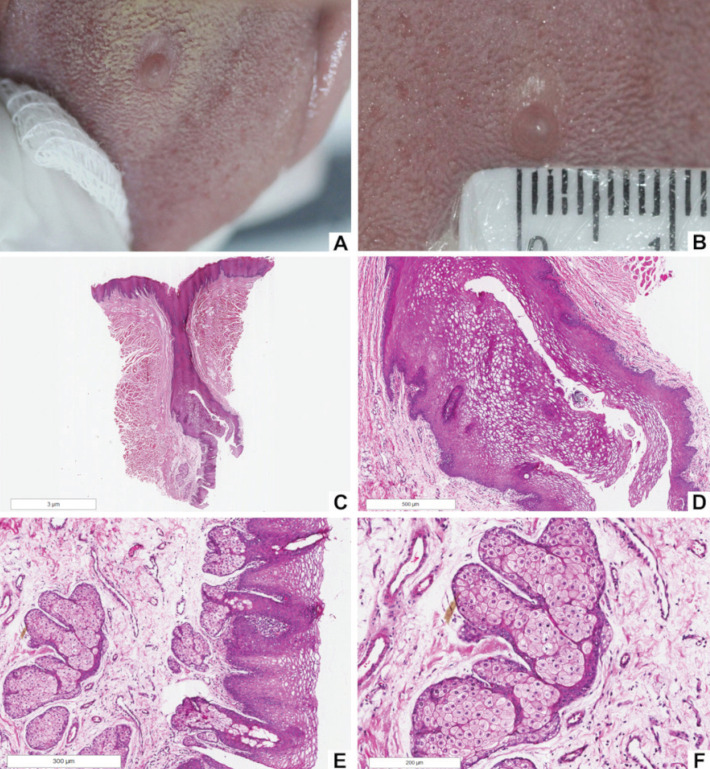
Clinical and histopathological aspects. Sessile papule, measuring 0.4 cm x 0.3 cm. The surface was smooth and the lesion was normochromic with a whitish center (A and B). Histological section stained with hematoxylin and eosin (H&E), showing oral mucosa covered by keratinized squamous epithelium. The center of the lesion presents an invagination of the epithelium into the connective tissue, forming a ductal structure covered by squamous epithelium (C). Squamous epithelium lining of the central duct (D). Ducts from the sebaceous glands connected to the central duct (E). Normal sebaceous glands (F).

On intraoral physical examination, there was a firm sessile papule, measuring 0.4 cm x 0.3 cm in diameter. The lesion was normochromic with a whitish center, had a smooth surface, and was located in the middle third of the dorsum of the tongue, in the midline (Fig. 1A,B).

An excisional biopsy with a 4 mm punch was performed. Histopathological examination showed parakeratinized squamous epithelium, with areas of orthokeratin and papillary atrophy. The center of the lesion presented an invagination of the epithelium into the connective tissue, forming a ductal structure covered by stratified squamous epithelium (Fig. 1C,D). The deeper areas had normal well-differentiated sebaceous glands (Fig. 1F), with ducts connected to the central duct (Fig. 1E). The connective tissue was dense, and deeply there was skeletal striated muscle and adipose tissue. The diagnosis, based on clinical and histopathological findings, was sebaceous choristoma.

## Discussion

The presence of scattered sebaceous glands in the oral mucosa or within the wall of cysts is not uncommon (3,7). However, these structures are rarely found in the dorsum of the tongue, with only 11 cases (5-7,9-13) reported in the English literature, and, of these, four were located in the midline (9-12). Most cases occurred in male patients and the age ranged from one month to 73 years (5-7,9-13). In only two of these cases, the lesion was congenital (5,6). Clinically, oral choristomas of sebaceous glands are firm nodules with 0.2 to 2 cm in diameter. They are normochromic to brownish color and are usually asymptomatic (Table 1,  Table 1 cont.,  Table 1 cont.-1,  Table 1 cont.-2). Six cases were associated with melanin pigmentation (5,10,13,14), and the only patient in the literature had multiple lesions (5).

**Table 1:**
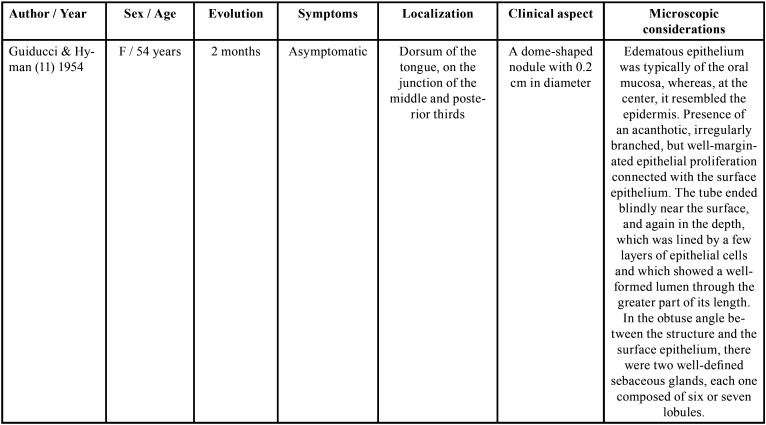
Clinical and histopathological characteristics of sebaceous choristomas in the tongue.

**Table 1 cont.**
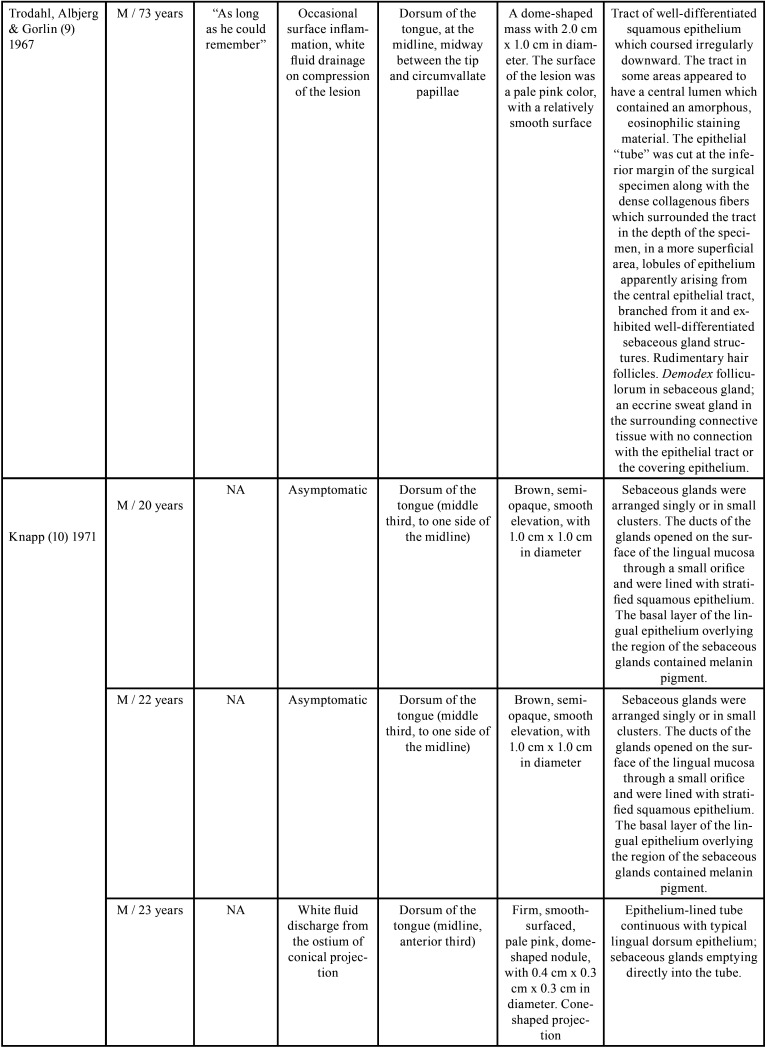
Clinical and histopathological characteristics of sebaceous choristomas in the tongue.

**Table 1 cont.-1:**
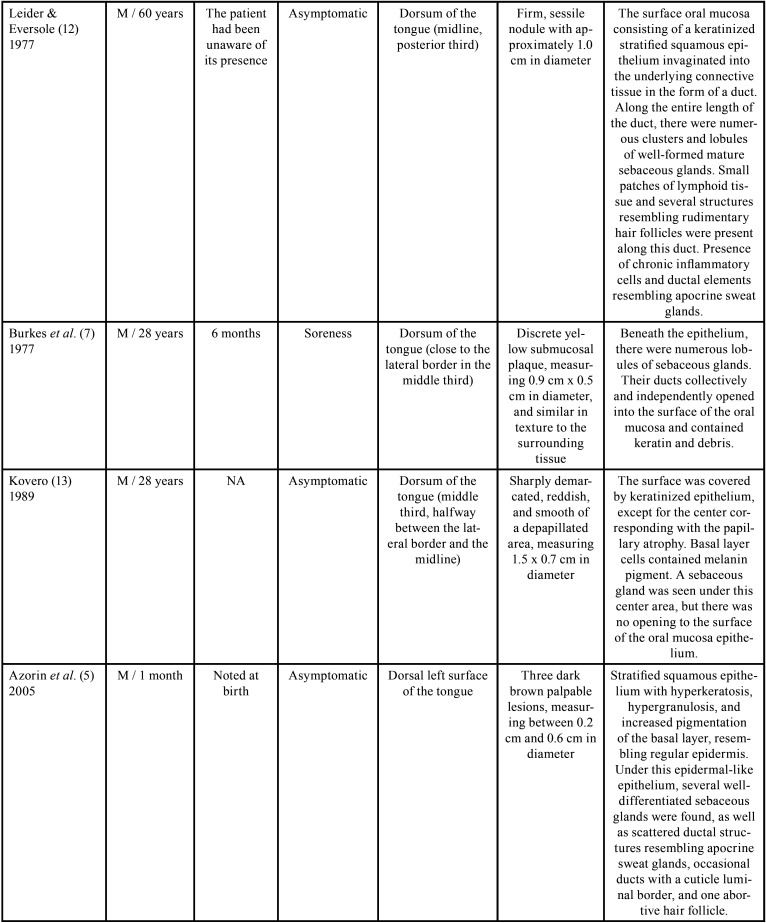
Clinical and histopathological characteristics of sebaceous choristomas in the tongue.

**Table 1 cont.-2:**
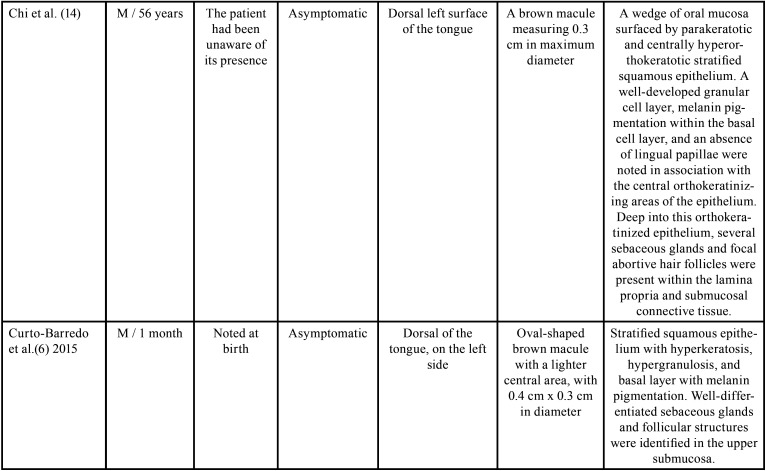
Clinical and histopathological characteristics of sebaceous choristomas in the tongue.

The first report of the presence of sebaceous glands in the tongue was made by Guiducci & Hyman (11), in 1954, who described the presence of a nodule located in the posterior third of the tongue, in the midline. The diagnosis was ectopic sebaceous gland, and the authors suggested that it could be the result of entrapment of epidermal elements of the pharynx in the tongue, such as pilosebaceous primordia and sweat glands during embryonic development (11). However, Knapp (10) suggested that, since sebaceous glands occur in the oral mucosa in almost 80% of the population, they should not be considered as ectopic glands, but as a sebaceous nevus.

Later, Leider *et al*. (12) described a similar sessile nodule, in the same location, close to the foramen cecum, and called it sebaceous choristoma. For these authors, the term choristoma would be more appropriate, as it refers to a growth of microscopically normal tissue in an abnormal anatomical site (12). The histopathological examination of this lesion revealed a stratified squamous epithelium covered by keratin, with a layer of cells with prominent granules and a moderately thick layer of spinous cells (12). The central region of the epithelium presented an invagination into the connective tissue in the form of a long duct, which was lined with keratinized stratified squamous epithelium (12). Moreover, along with the duct, aggregates of lymphoid tissue and well-differentiated mature sebaceous glands were present (12). Except for the presence of lymphoid tissue, these histopathological findings were similar to the present report. However, clinically, their location was posterior. The authors considered this lesion an abnormality of the thyroglossal duct because of this posterior location and lymphoid follicles (12).

There is another case in the literature also attributed to remnants of the thyroglossal duct (10), which was a normochromic papular lesion with a cone-shaped projection located in the anterior third in the midline of the tongue (10). According to the author, the lesion could be the result of a variation in the positioning of the thyroglossal duct (10). Clinically, the lesion also had a central ostium, but it drained a white fluid (10). Exploration with a probe showed that the opening led to a duct, which extended into a plane parallel to the dorsum of the tongue, with a distance of 3 cm (10). The histopathological examination verified that the sebaceous gland ducts were also connected to the central duct. However, there were not lymphoid follicles, as in the present case (10).

The remnants of the thyroglossal duct are one of the most common congenital masses of the head and neck in childhood (15). Embryologically, the development of the thyroid gland begins at the end of the third week of intrauterine life, as a proliferation of endodermal cells on median surface of developing pharyngeal floor (foramen cecum) from 1st and 2nd pharyngeal arches (16). This proliferation migrates to the neck and, along this descending path, a narrow structure is formed called the thyroglossal tract or duct (12), maintaining a union with the base of the tongue (16). Usually, the thyroglossal duct suffers atrophy and disappears by the end of the eighth intrauterine week (12). However, the point of origin of this tract usually remains visible, as the foramen cecum, which is seen as a small depression in the posterior region of the dorsum of the tongue, in the midline (12). Anatomical variations have also been reported in thyroglossal duct cysts (17,18). The sebaceous gland entrapment and the anterior displacement of this anatomical structure to the medium portion of the tongue, during embryogenesis, could also explain the occurrence of the lesion in the present report. The characteristics that support this hypothesis are: congenital lesion, midline location, trapping of epithelial tissue, which could be a remnant of the thyroglossal duct, and the presence of a central duct. However, no lymphoid tissue was found in the vicinity of the lesion, as it is commonly associated with the vestigial thyroglossal ducts (11,12).

Trodahl & Gorlin (9) described a similar case to the one presented in this paper. It was a dome-shaped mass with 2 cm in diameter, with a smooth and normochromic surface, located on the middle portion of the tongue, in the midline. The lesion was firm on palpation and asymptomatic. However, it sometimes became inflamed and a white fluid drained when pressure was applied (9). The histopathological examination showed, in a more superficial area, lobes of well-differentiated glandular structures, apparently originating from the epithelium (9). However, unlike the present case and the cases mentioned before (10,12), in which the sebaceous glands were connected to a central duct, the presence of an eccrine sweat gland in the surrounding connective tissue with no connection with the epithelial tract or with the lining epithelium was identified (9). Moreover, the authors reported that, although some areas resembled rudimentary hair follicles, no well-defined pillar element was found (9). Other reports of choristomas with glands without communication to the surface of the epithelium (13), as well as glands flowing into independent openings on the dorsum of the tongue (7,10) were also described in the literature.

In 2005, Azorin *et al*. (5) described a case of a newborn with three congenital pigmented lesions on the tongue. The histopathological examination showed an abrupt replacement of the typical superficial epithelium by stratified squamous epithelium, showing hyperkeratosis, hypergranulosis, and intense melanin pigmentation within the basal layer cells (5). Below the epithelium, several sebaceous glands, an abortive hair follicle, and ductal structures similar to apocrine sweat glands were observed (5). As normal skin elements were found in an abnormal location, and because the skin appendices are embryologically derived from the epidermis, the authors attributed the terms cutaneous or epidermal choristoma to these lesions (5). Furthermore, they suggested the possibility that sebaceous choristomas and lingual congenital melanocytic macules could represent different expressions of the same disorder (5). Other reports of tongue epidermal choristomas were also described (5,14).

Despite being rare, sebaceous choristomas should also be considered in the differential diagnosis of tongue abnormalities or diseases. Although the pathogenesis is not well understood, the present report, as it is a congenital case in the midline, reinforces the hypothesis of a disorder with embryological origin and a possible relationship with thyroglossal duct remnants.
